# Steel Sheets Laser Lap Joint Welding—Process Analysis

**DOI:** 10.3390/ma13102258

**Published:** 2020-05-14

**Authors:** Hubert Danielewski, Andrzej Skrzypczyk

**Affiliations:** Faculty of Mechatronics and Mechanical Engineering, Kielce University of Technology, Al. 1000-lecia P.P. 7, 25-314 Kielce, Poland; tmaask@tu.kielce.pl

**Keywords:** laser welding, steel sheets, numerical simulation, lap joints, mechanical properties, microstructure analysis

## Abstract

This article presents the results of steel-sheet lap-joint-welding using laser beam radiation. The use of a laser beam and keyhole effect for deep material penetration in lap joint welding was presented. Thermodynamic mechanism of laser welding is related to material properties and process parameters. Estimation of welding parameters and joint properties’ analysis was performed through numerical simulation. The article presents a possibility of modeling laser lap-joint welding by using Simufact Welding software based on Marc solver and thermo-mechanical solution. Numerical calculation was performed for surface and conical volumetric heat sources simulating laser absorption and keyhole effect during steel sheet welding. Thermo-mechanical results of fusion zone (FZ), heat-affected zone (HAZ) and phase transformations calculated in numerical simulation were analyzed. The welding parameters for partial sealed joint penetration dedicated for gas piping installations were estimated from the numerical analysis. Low-carbon constructional steel was used for numerical and experimental analyses. A trial joint based on the estimated parameters was prepared by using a CO_2_ laser. Numerical and experimental results in the form of hardness distributions and weld geometry were compared. Metallographic analysis of the obtained weld was presented, including crystallographic structures and inclusions in the cross section of the joint.

## 1. Introduction

Welding methods are based on the thermal effect of melting and crystallization process. Conventional methods use electric arc as a heat source. Alternative to gas metal arc welding (GMAW) are beam welding methods [1,2], in which the concentrated energy of focused electrons or photons achieves high power density. The energy distribution factor allows high-speed welding, and the quantity of thermal energy absorbed in the materials is low. Electron beam welding (EBW) has high energy distribution, but it has to be performed in a vacuum, which is problematic in some welding applications. Laser beam welding (LBW) is an alternative technology in which a high power density of a focused photon beam shielded by inert gas can be used for numerous types of joining applications [3,4]. The keyhole effect in LBW enables deep penetration of the welded material. Moreover, laser beam penetration is possible through more than one material. Rapid development of laser technology has determined LBW for use in advanced joint configuration. Currently, researchers are focusing on welding dissimilar materials, where low-carbon and austenitic steels are welded, and some works are related to joining advanced aluminum, nickel and titanium alloys [5,6,7,8,9]. Nonconventional joining methods, such as laser welding in butt, lap and T-joint configurations, are also being widely studied [10,11,12,13,14]. Numerical analysis of the laser welding process has been undertaken, including lap joints, for a wide range of materials by many researchers [15,16,17,18]. However, laser lap welding of low-carbon steel has been reported in only a few works concentrated mostly on numerical analysis or joints properties [19,20,21,22,23,24]. The works cited above lack a more comprehensive study of lap joints, laser beams applied to the welding of commonly used constructional steel, or analysis based on mechanical and metallographic study, supported by numerical simulation. The selection of manually programmed laser welding parameters is problematic and requires performing a number of trial joints and an experienced operator. It thus seems reasonable to use some aided methods for supporting welding parameters estimation, such as analytical method, where thermal conduction calculation is based on solving the moving heat-sources equation proposed by Rosenthal [9]. Evolution of analytical computation relies on improving the mathematical description of heat sources. Solving the moving-heat-sources equation enables the use of the thermal distribution to estimate the shape of weld geometry. Analytical solution allows for the estimation of welding parameters in simple cases. In applications such as lap joints of steel sheets, it is more complex and requires using numerical methods [25,26,27]. 

Numerical simulation of welding process can be performed by using dedicated software such as SYSWELD or Simufact Welding or advanced multiphase heat-mass flow programs such as ANSYS with FLUENT module. Calculations are based on the Finite Elements Method (FEM) and solver engine. CAD geometry is discretized, and finite elements mesh is generated. During discretization of the area where significant heat effect can occur, FE refinement is performed. Welding simulation requires defining heat-source dimensions and heat-energy volume related to welding parameters [28,29,30,31,32,33]. If we consider thermo-mechanical simulation, in addition to the results of temperature distribution, a stress–strain analysis can be obtained. Considering lap welding, material properties in the upper plate of joint will differ from those in the lower plate. An analysis of this phenomenon is presented in this article. Properties of stress–strain distribution in the welded material are related to thermo-physical material properties, temperature distribution, heat expansion coefficient and phase transformation, changing in time. Defining these properties is complex; nevertheless, using numerical computation accurate estimation of the results is possible. 

When performing a welding simulation, we must remember that, no matter how accurate, the results obtained are just estimations. The quality level depends on the defined boundary conditions and programmed welding parameters; therefore, experimental verification is required. The welding process requires a shielding atmosphere of inert gas. In order to confine ionization effect, helium is recommended as a reference gas. In the case of a sealed lap joint with partial penetration of the lower plate, no shielding atmosphere for the weld root is needed. However, the space between the plates contains some oxygen and may cause inclusions and welding defects; therefore, metallographic analysis was performed in this study. A programming numerical model verification stage needs to be considered, especially for defining some process properties, such as heat-source efficiency and energy distribution (TEM), related to the laser type, which must be included in the simulation [34,35,36,37,38]. The programmed heat-source dimension can be verified by comparing the simulated weld geometry with the trial joint.

In this paper, a numerical-simulation-aided analysis of laser welding of sealed steel sheets’ lap joints is presented. 

## 2. Methodology

### 2.1. Materials

Low-carbon constructional steel S235JR in the form of 2.5 mm thick steel sheets was used as a base material for both the simulation and trial joint welding. The commonly used low-carbon steel sheets were welded in lap joint configuration, using an advanced heat source in the form of a laser beam. The S235JR is unalloyed steel with carbon content up to 0.2% and a trace amount of other alloying elements (Table 1).

The structure of S235JR steel is typically ferritic–pearlitic. Low-carbon content and the trace amount of alloying elements reduce steel hardening, though some strengthening effect may occur via steel phase transformations. Thermo-physical properties of the material affect heat expansion and welding results (Table 2). The S235JR steel has high thermal conductivity, with the phase transformation temperature of 725 °C for AC_1_ and 863 °C for AC_2_ [39,40].

### 2.2. Numerical Simulation

**Figure 1:**
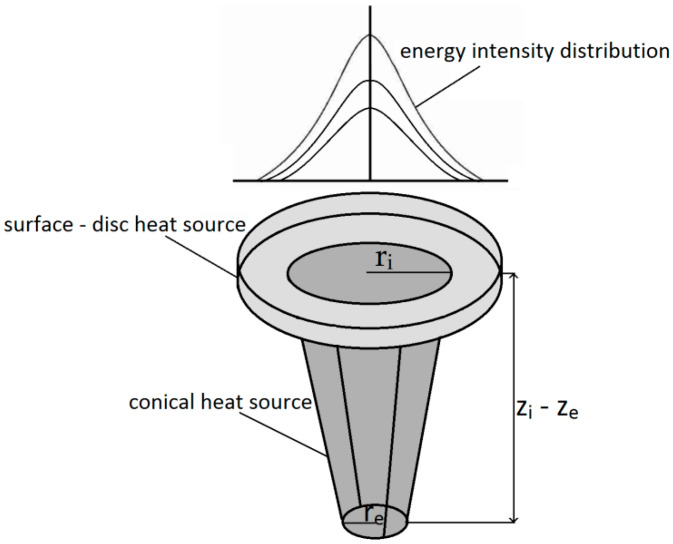
Heat-source model used in laser-welding simulation.

A numerical simulation of laser welding generally uses two types of heat-source models simulating laser interaction with the material: a surface heat source and a conical heat source. The surface-heat-source model (disc shape) is more accurate and is used for simulating laser energy absorption by steel sheets’ surface (in some applications, for conduction welding solutions) [41]. The conical heat source is dedicated to simulating the keyhole effect and is related to energy being directed inside the material through the keyhole walls. Simufact Welding software (Simufact Engineering GmbH, Hamburg, Germany) uses a combination of surface and conical volumetric heat sources with uneven energy intensity distribution (Gaussian parameter), as shown below (Figure 1). 

Conical volumetric heat source with the Gaussian distribution can be described by the following equation:

(1) $$Q\left(x,y,z\right)=Q_{0}\exp\left(-\frac{x^{2}+y^{2}}{r_{0}^{2}\left(z\right)}\right)$$

Moreover, $r_{0}\left(z\right)$ is defined as follows:

(2) $$r_{0}\left(z\right)=r_{e}+\frac{r_{i}-r_{e}}{z_{i}-z_{e}}\left(z-z_{e}\right)$$

where $Q_{0}$—maximum volumetric heat flux density$;\;r_{i}-r_{e}$—upper and lower conical radius dimension; $z_{i}-z_{e}$—conical heat source depth; and *x*, *y*, *z*—coordinates of heat source.

The numerical simulation was performed by using Simufact software with a Marc solver. The program is dedicated to welding applications; however, some physics phenomena, such as solidification, are simplified. This phenomenon is solved by using the assumption that the latent heat is uniformly released within the solidus and liquidus temperature range, where the solver uses the modified specific heat to model the latent heat effect based on material experimental data calculated by using JMatPro. Thermal conductivity is the dominant heat-transfer method; therefore, the governing equation is based on this phenomenon. Based on Fourier’s law, three-dimensional heat conduction is given by the following governing equation:

(3) $$\rho c\left(T\right)\frac{\partial T}{\partial t}=\frac{\partial }{\partial x}\left(k\left(T\right)\frac{\partial T}{\partial x}\right)+\frac{\partial }{\partial y}\left(k\left(T\right)\frac{\partial T}{\partial y}\right)+\frac{\partial }{\partial z}\left(k\left(T\right)\frac{\partial T}{\partial z}\right)+q_{v}$$

where *c*(*T*)—temperature dependent specific heat capacity; *k*(*T*)—temperature dependent thermal conductivity; $q_{v}$—volumetric internal energy; *x, y, z*—space coordinates; *T*—temperature; ρ—density; and *t*—time.

The simulation includes the convection effect and uses the Petro–Galerkin convection–diffusion model and nodal velocity vectors, as shown below.

(4) $$\frac{\partial T}{\partial t}+v\cdot \nabla T=\nabla \cdot \left(\kappa \nabla T\right)+Q$$

where v—nodal velocity vector; *T*—temperature; κ—diffusion tensor; and *Q*—source term.

The obtained numerical model accounts for convection but does not account for surface tension or the Marangoni effect; therefore, some differences in heat transfer compared to the experimental process will occur, reflected in the fusion zone shape.

**Figure 2:**
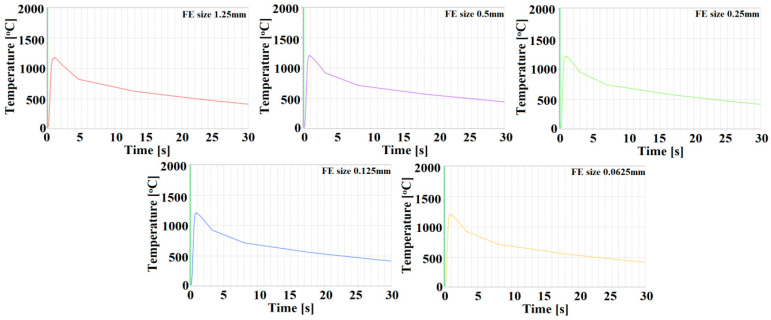
Graphs showing temperature changes for different FE mesh size.

In the boundary conditions, a rigid restraint for welded elements, using fixed geometry, was programmed (Figure 2). Two 2.5 mm thick sheets were meshed by using finite elements hexahedral in shape. A preliminary research of mesh convergence was carried out, where welding simulations were performed with the same process parameters and different mesh sizes, starting with FE size equal to 1.25 mm, and then 0.5, 0.25 and 0.125 mm up to 0.0625 mm. For 0.0625 and 0.125 mm, no significant differences in the weld or HAZ geometry were observed; however, some differences between 0.125 and 0.25 mm were detected. In order to confirm mesh convergence, a study of temperature distribution was carried out. For all mesh sizes, measurement point, placed 2.5 mm from weld axis (approximately center of HAZ) were sets, and temperature changes shown in graph form were compared (Figure 2). The graphs in Figure 2 show similar results as the HAZ geometry analysis.

Therefore, in order to save simulation time, a general FE size was programmed as 0.25 mm. In order to obtain more accurate and realistic results, a refinement procedure with FE size equal to 0.125 mm was performed near the weld zone (at the temperature exceeding 400 °C). Sheets of S235JR low-carbon constructional steel were selected for the simulation (Table 2). The material multiphase library allowed the calculation of the overall thermo-mechanical joint properties.

The performed research assumed keyhole welding and deep material penetration; however, in the first stage before the keyhole effect appears, laser beam reflectivity from the metal surface is high, and when the heat-source efficiency is programmed, this phenomenon must be included. Therefore, for the simulation of CO_2_ laser welding, the heat source efficiency coefficient was assumed as 0.77.

The geometry of the heat source is related to the focal length and focusing power of welding optics, and for the performed research, a single spot spherical mirror with a focal point diameter of 0.3 mm and a focal length equal to 200 mm was used. The disc-shaped heat source with a radius equal to 0.65 mm and a depth of 0.2 mm, and the conical heat source with the upper radius of 0.5 mm and the lower radius of 0.2 mm and the depth of 4 mm were programmed. Geometry of the heat source (HS) is related to welding optics. Nevertheless, some calibration for more accurate results is required, and test welding at the speed of 1 m/min and output power equal to 1 kW was performed. Comparison with the simulation results showed some discrepancy, and the heat-source geometry was adjusted by reducing the HS radius by approximately 10% and Gaussian distribution parameter from 2.9 to 2.8 [42,43].

**Figure 3:**
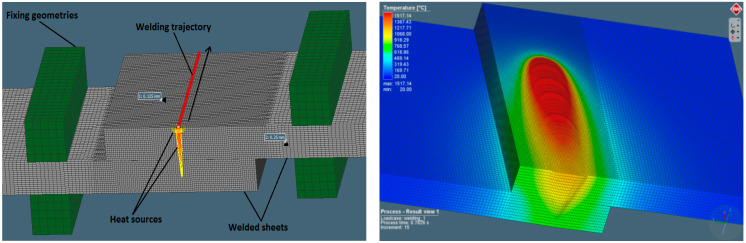
Laser-welding simulation model and the weld forming during the lap joint welding process.

Numerical calculation of the laser lap-joint welding using a single pass process simulated by the heat source moving through steel sheets was carried out (Figure 3). Welding simulations with constant speed rate of 1 m/min and changing output power from 1 to 5 kW, changing with a step of 0.5 kW, were performed until the assumed sealed lap joint geometry was obtained. Phase transformation requires cooling time, so for welding equal to 1.2 s, the time for the complete process was programmed as 30 s. The simulation process was performed on a Dell PC class station with the i7 processor and 64 GB RAM, and the simulation calculation time was about 26 h.

### 2.3. Experimental Procedure

**Figure 4:**
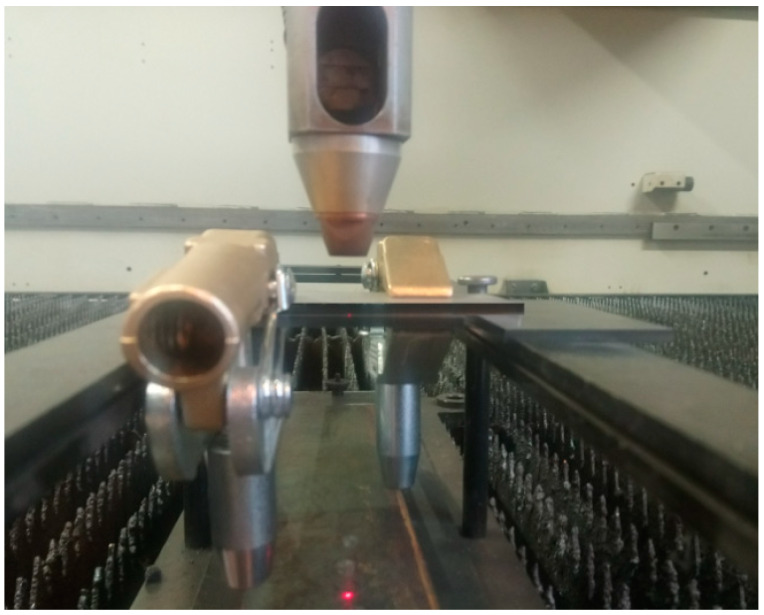
Laser-welding station with lap joint low-carbon steel configuration.

Verification of the numerical model was performed by welding the trial joint with parameters estimated at the simulation stage (laser power equal to 4 kW, with the welding speed of 1 m/min). For the configuration of the sealed steel sheet lap joint with partial penetration, welding conditions equal to those defined in the numerical simulation were established. In order to reduce the plasma ionization effect, helium as a shielding gas, with a flow rate equal to 20 L/min, was used. The welding process was performed with a CO_2_ laser Trumpf TruFlow 6000 integrated with a 6 axis LaserCell 1005 work station (Figure 4).

The welding head with a focal length of 200 mm and coaxial shielding gas delivery system was used. The focal point was placed on the upper steel-sheet surface, and the welding of the trial joint was performed. The upper sheet was welded through, and the lower plate had the fusion zone approximately in the middle of its thickness. Therefore, the assumed lap joint partial penetration of two 2.5 mm thick S235JR steel sheets was obtained [44,45,46].

**Figure 5:**
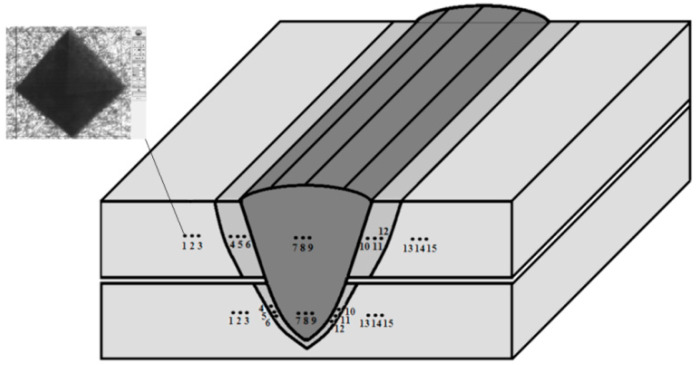
Hardness test point distributions in the lap joint.

Weld strength characteristics were defined by the mechanical properties of the obtained joint. The weld properties were investigated by using destructive tests. The hardness test was carried out according to PN-EN ISO 6507-1 standard, using an Innovatest Nexus 4303 machine [47], and the hardness test point distribution for the steel sheets’ lap joint is shown below (Figure 5).

The three-point test was performed according to the standards for all characteristic zones: base material (BM), heat-affected zone (HAZ) and fusion zone (FZ) in the upper and lower plates.

**Figure 6:**
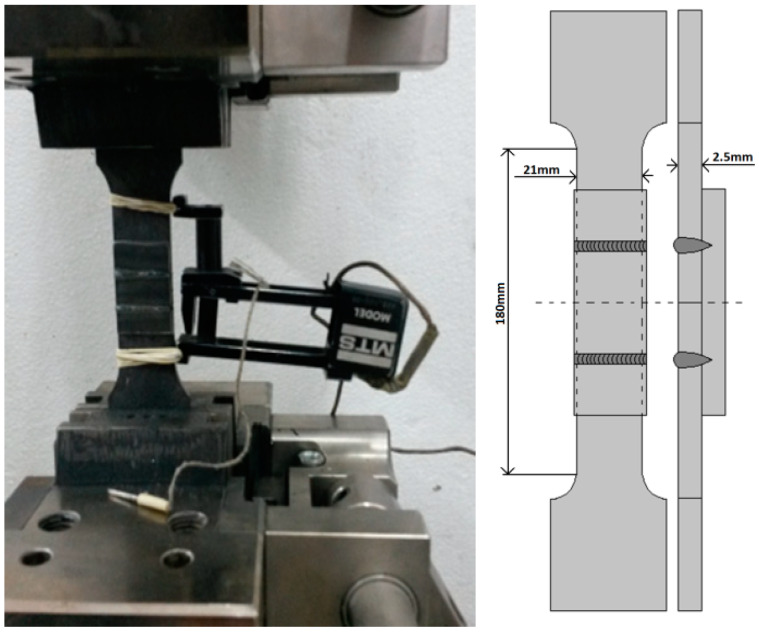
Tensile-strength test stand and specimen scheme.

The material hardness resulting from the crystallographic structure of the welded material affects joint-strength characteristics. Moreover, the properties are related to the thermal cycles and chemical composition of the material. In order to investigate the joint-strength properties, the tensile-strength test was carried out, using an MTS-100 testing machine (MTS Systems Corporation, Eden Prairie, MN, USA) (Figure 6) [48].

The specimen was prepared by welding two upper plates to a lower plate, with the same process parameters as presented in the scheme (Figure 6). In this configuration, stretching, as well as shearing phenomena, will occur during the test. To confirm the obtained joint properties, an additional test of a specimen welded with the same parameters was performed. No-uniaxial complex-force distribution will certainly affect the test results. Nevertheless, the bonding force of welded sheets in the sealed lap joint will be related to the weld properties; therefore, metallographic analysis is required.

Metallographic tests were carried out according to PN-EN ISO 17639 [49]. A microscopic and macroscopic test, using a HiroxKH-8700 confocal digital microscope (Hirox Co Ltd., Tokyo, Japan), was performed, in order to investigate the crystallographic structure of the welded material.

The obtained welded lap joint was analyzed. The low-carbon S235JR steel is not typical hardening steel; nevertheless, phase transformation during laser welding affects the material, and the structure changes. A visual microscopic test was carried out to confirm the weld quality and to detect any defects. The crystallographic structure in the upper and lower plate after welding was investigated [50,51].

Laser welding of overlap joints is complex. Thin interspace between welded sheets can affect porosity and oxides’ formation. To confirm the uniform structure of the weld, the qualitative and quantitative analysis was carried out, using energy-dispersive X-ray spectroscopy with a scanning electron microscope JSM-7100F (JEOL Ltd., Tokyo, Japan).

## 3. Results

### 3.1. Simulation Analysis Results

**Figure 7:**
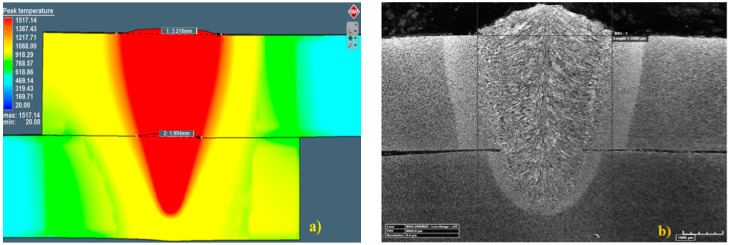
Laser lap-joint welding results from (**a**) simulation, (**b**) experiment.

**Figure 8:**
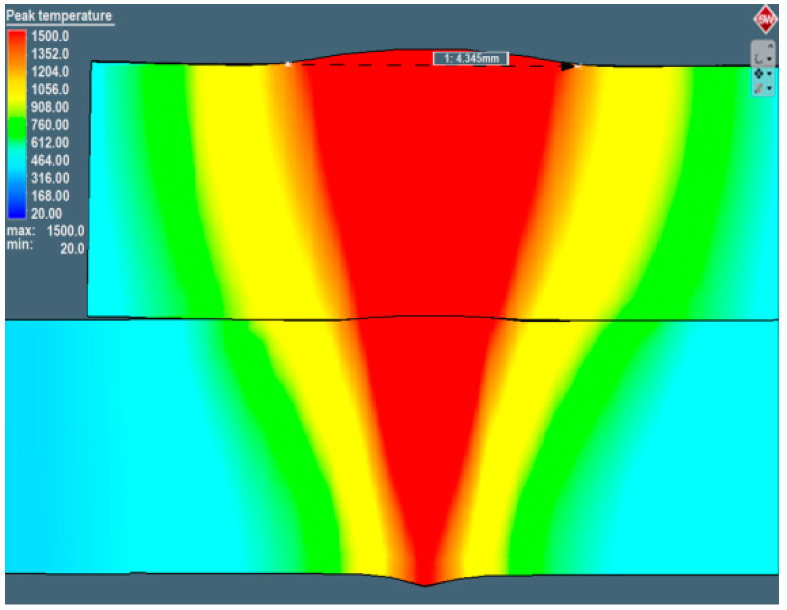
Laser lap-joint welding results for complete penetration.

Laser-welding parameters were estimated by using numerical simulation analysis. Welding-simulation parameters with a speed rate of 1 m/min, output power 4 kW, heat source efficiency of 0.77 and the Gaussian parameter of conical and surface heat source equal to 2.8 were programmed (according to laser TEM01* mod). According to those parameters, the assumed partial penetration in the joint was obtained. Results of the simulation showed that the output power equal to 4 kW provided the partial welding penetration (Figure 7a). Further analysis showed that, by increasing the output power by 0.5 kW, the complete penetration was obtained (Figure 8). For the assumed geometry of heat sources and programmed boundary conditions, partial penetration of the sealed lap joint was achieved and considered in further investigations. The thermo-mechanical simulation, which took into account phase transformation, gave realistic results of the welding process, with a convex face of the weld and material deformation obtained by a solver mechanism. The recalculation of the contact tolerance or remeshing of the distorted region was performed (Figure 7a and Figure 8). Macroscopic examination of the cross-section of the trial joint was performed by using a confocal digital microscope HiroxKH-8700 with a magnification of ×35 (Figure 7b).

**Figure 9:**
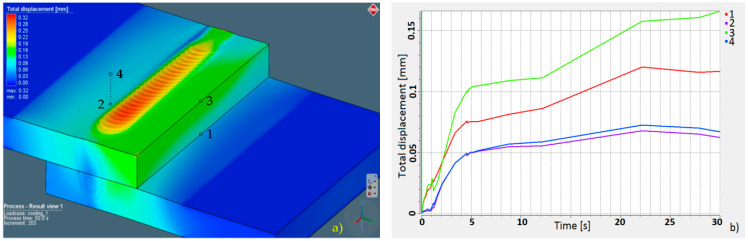
Total displacement: (**a**) distribution map and (**b**) displacement chart according to measurement points.

**Figure 10:**
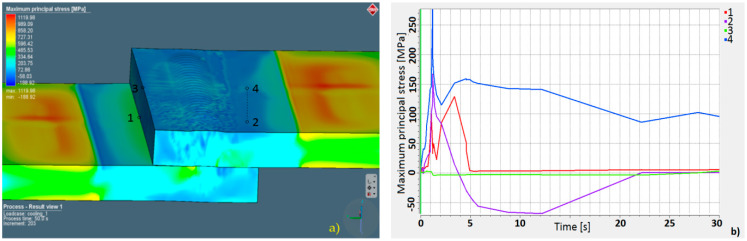
Maximum principal stress: (**a**) distribution map and (**b**) stress chart according to measurement points.

Parameters estimated in the numerical simulation gave similar results: The face of the weld in the simulation was 3.22 mm in width, and in the experimental welding, it was 3.38 mm (for the complete penetration this value was equal to 4.35 mm). Moreover, the weld width in the overlap area for the simulation was equal to 1.99 mm, and it was 2.05 mm for the trial joint. The depth of the obtained welds was 4.33 mm for the trial joint and 4.38 for the simulation. Macroscopic analysis showed differences in heat expansion; the HAZ in the upper plate was wider. This effect is related to dumping and energy decrement during the penetration of the lower sheet. Although the welded surfaces adjoin each other, the spot size of the laser beam on the lower sheet during surface penetration was bigger, indicating lower power density. This phenomenon was also related to the heat-expansion direction. In the upper plate, heat expanded only in the XY direction, and in the lower sheet, it expanded in the XYZ direction. The macroscopic examination confirmed the accuracy of the simulation results, and it was possible to perform further numerical analyses. A thermo-mechanical simulation and a stress–strain analysis were carried out. The overall calculated displacement (Figure 9a) was 0.32 mm, with maximum principal stress (Figure 10a) of 1120 MPa. The changes in the total displacement (Figure 9b) and maximum principal stress (Figure 10b) were recorded against the defined measurement points. Higher displacement values occurred in the steel sheet plate edge (at points 1 and 3). The higher maximum principal stress concentration was measured on the opposite side of the weld (at points 2 and 4).

### 3.2. Analysis of the Results for Properties

**Figure 11:**
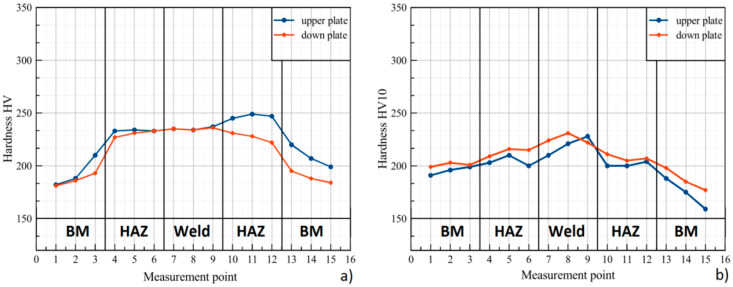
Results of hardness distribution in cross-section from (**a**) simulation and (**b**) experimental welding.

Numerical simulations with phase transformation allowed for the calculation of material phase change and hardness distribution. As set forth in the standards, the three-point test was performed for all characteristic zones in the upper and down plates, in the cross-section, as defined in measurement point distribution (Figure 5). Results from the simulation are shown in Figure 11a, and the results obtained for the trial joint are shown in Figure 11b.

The hardness test results show strengthening in the weld and HAZ. The hardness distribution obtained from the simulation differs from the results measured on the trial joint. The calculated hardness values are greater than those of the welded materials. Hardness simulated for the weld zone does not exceed 240 HV (maximum value is equal to 237 HV). Discrepancies between the upper- and lower-plate values can be observed. In the HAZ for the upper plate, it takes the value from 233 to 249 HV, and for the lower plate, from 222 to 233 HV. The experimental hardness values (trial joint) are lower than the calculated ones. The highest measured value is 231 HV10 and occurs in the weld. In HAZ, the hardness value ranges from 200 to 218 HV10, and in the BM, it is from 160 to 200 HV10. Differences between measured and simulated values amount to 6 HV in the weld and 31 HV in HAZ. Despite the high accuracy of the obtained weld geometry, the results from the simulation and experiment vary. The discrepancies depend on phase-transformation phenomena (temperature gradient and chemical composition of welded material) and affect the calculated and measured hardness values [52,53]. According to PN-EN ISO 15614-11, the maximum allowable limit of Vickers hardness HV10 after welding is 350. Neither calculated nor measured hardness exceeded the allowed value. Therefore, no additional post-weld heat treatment was carried out in the simulation or on the trial joint.

**Figure 12:**
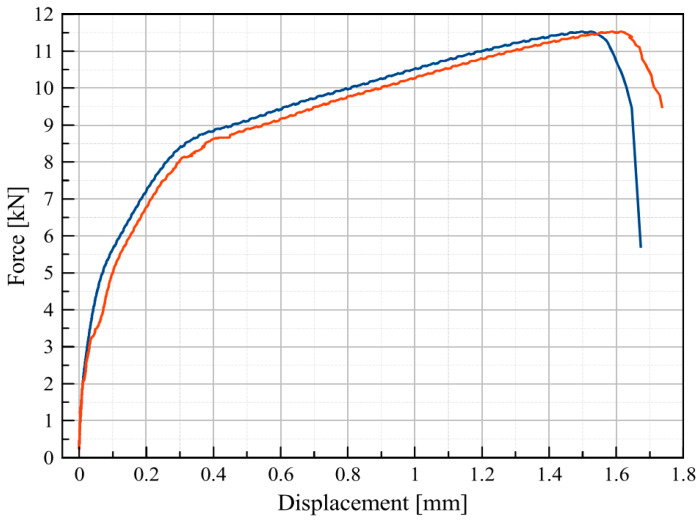
Tensile strength test results.

Material-strength characteristics change during the welding process, depending on the phase transformation phenomena, and are different for the fusion zone, heat-affected zone and base material. The hardness test showed differences in the trial joint properties relative to the simulation results. Therefore, in order to prove the high quality of the joint obtained by using the estimated parameters, a static tensile test was performed. The properties of the obtained joint were confirmed by performing an additional verifying test of joint strength. Manufactured specimens were stretched by increasing the loading force until failure at the tensile test rate equal to 2 mm/min. The results of the tensile test were compiled as a force vs. displacement graph (Figure 12).

The static tensile test results show failure of the lap joint at the maximum force equal to 11.5 kN for the first specimen, and at 11.49 kN for the other specimen. The strength of the obtained joint was 110 and 108 MPa, respectively. The tensile strength of BM was 360 MPa. The failure of both samples occurred along the fusion-zone line. The joint configuration (Figure 6) affected the measurement results. Tension and shear occurred, and the results obtained were mostly dependent on the weld strength [54,55,56,57].

### 3.3. Metallographic Analysis

**Figure 13:**
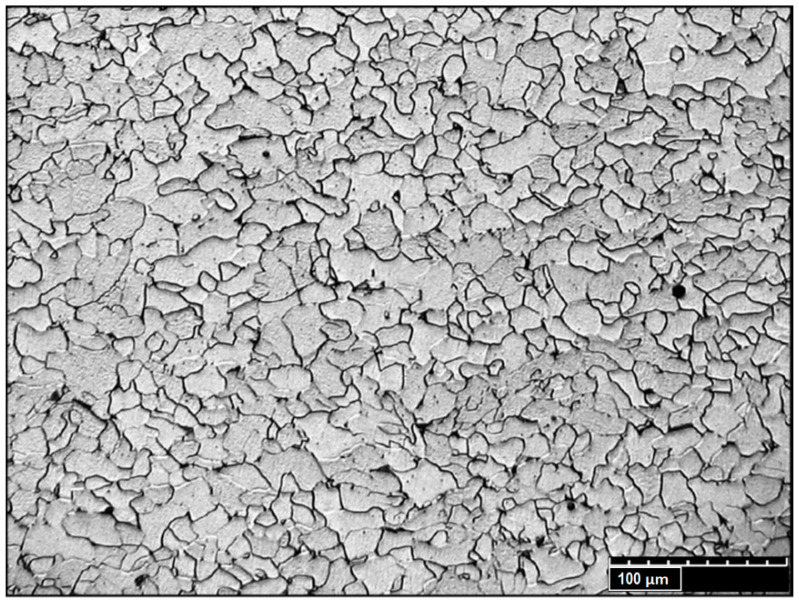
Microstructure of BM.

Optical and electron microscopes were used for crystallographic structure analysis. The structure of the base material (Figure 13) was examined by using a HiroxKH-8700 confocal digital microscope at a magnification of ×800. It showed a typical low-carbon structure. The microstructure of BM was characterized as fine-grained ferritic–pearlitic (dark areas: ferrite and bright: pearlite), with no explicitly shown banding, which is characteristic of S235JR steel.

The low-carbon S235JR steel is not a typical hardening material, but phase transformation during laser welding at the speed rate of 1 m/min affected the crystallographic structure. The microscopic examination showed a good-quality weld with no defects (Figure 7b).

**Figure 14:**
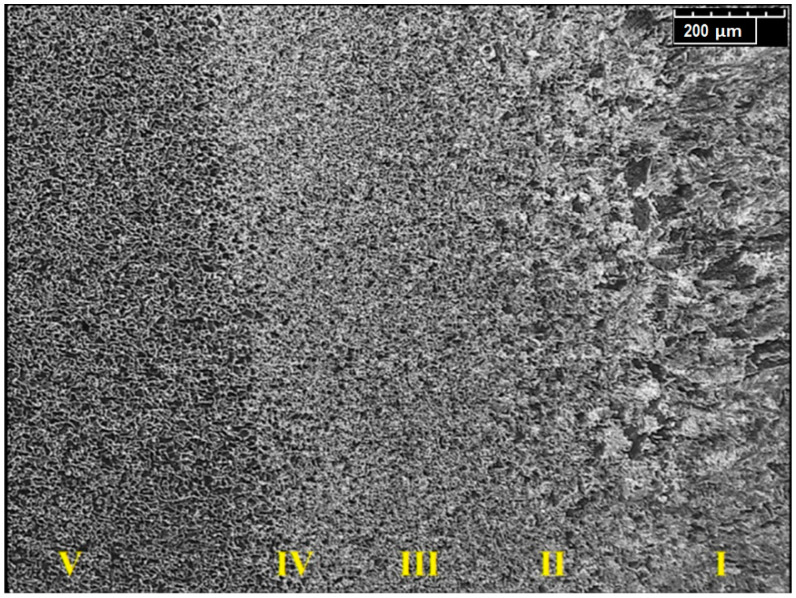
Microstructure of HAZ.

The HAZ structure analysis using an optical microscope with a magnification of ×140 was performed (Figure 14). In the weld interface, assuming a direction from the fusion (I) toward the BM (V), three characteristic areas were identified: II—overheated zone, III—normalization zone and IV—partial recrystallization zone.

**Figure 15:**
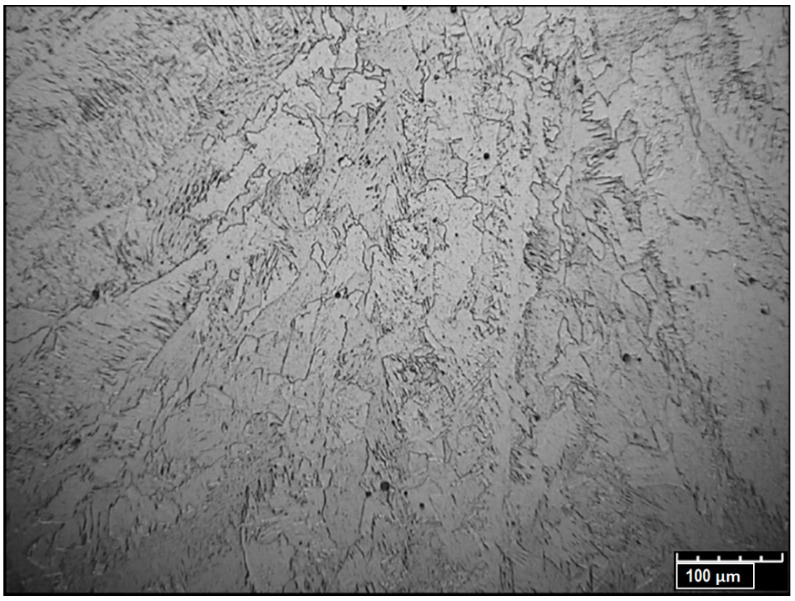
Microstructure of the weld.

The weld-structure investigation was performed by using an optical microscope at a magnification of ×400. The weld exhibited a coarse-grained dendritic structure (Figure 15), which is typical for the laser-welding process.

**Figure 16:**
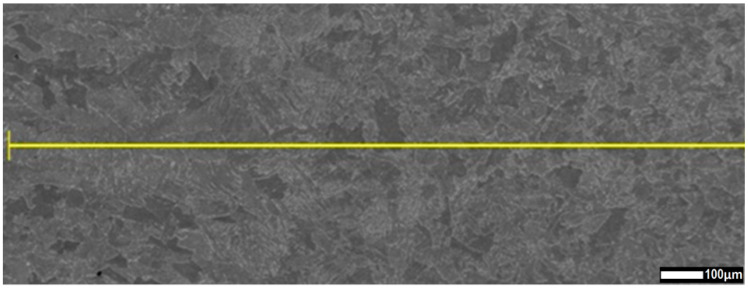
HAZ-to-FZ measurement line for chemical composition analysis.

**Figure 17:**
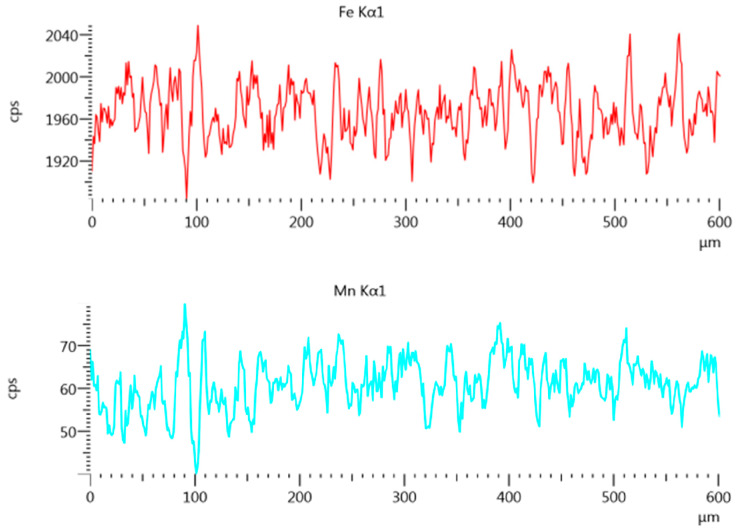
Ferrite and manganese content along the HAZ-to-FZ measurement line.

**Figure 18:**
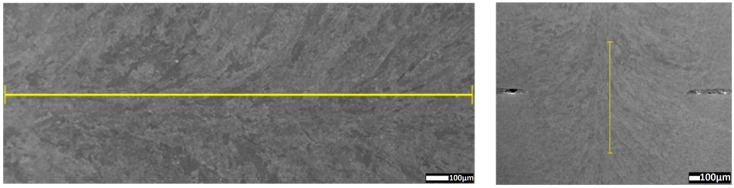
Measure line of fusion zone in overlap transition.

**Figure 19:**
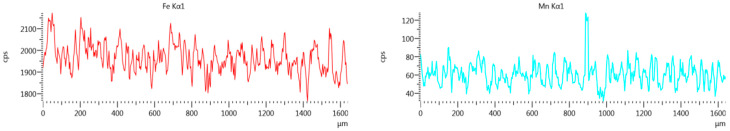
Ferrite and manganese amount along the overlap transition measurement line.

The structure, precipitations and oxides in the weld across the interspace line between the welded sheets were determined by using energy-dispersive X-ray spectroscopy performed on a JSM-7100F scanning electron microscope. The chemical composition in the cross-section of HAZ to FZ (Figure 16 and Figure 17) and weld (Figure 18 and Figure 19) was analyzed [58].

A chemical composition analysis of HAZ to FZ, based on ferrite and manganese distribution, was performed (Figure 16). A uniform mixture of ferrite and manganese was found across the measured line (Figure 17).

Using the ferrite and manganese distribution to identify fusion-zone uniformity, the weld in the overlap area was analyzed (weld transition) (Figure 18).

**Figure 20:**
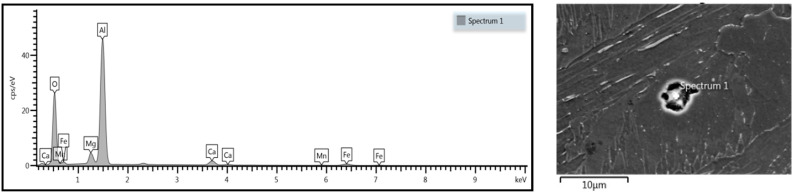
Spectroscopy analysis of identified aluminum oxide in the weld.

**Figure 21:**
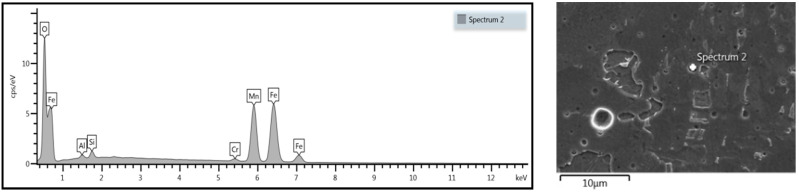
Spectroscopy analysis of identified oxide in the weld.

Uniform distribution of the measured elements showed a high mixing factor. Precipitation analysis of fusion zone was performed, and some inclusions were detected (Figure 20 and Figure 21). 

Analysis of weld transition zone revealed the presence of aluminum oxide (Figure 20). 

Further analysis revealed other oxides (Figure 21). In addition to aluminum oxide (Figure 20), manganese oxides in significant quantities were detected in the overlap fusion zone. No oxides or inclusions were found in the face and root of the weld. No impurities affecting the mechanical properties of the weld, such as phosphorus or sulfur, were detected. 

## 4. Discussion

The numerical simulation of laser welding in lap joint specimens was performed, and welding parameters for obtaining partial penetration for the sealed joint were estimated. According to simulation results, 4 kW of output power with a speed ratio equal to 1 m/min gave enough linear power density to obtain a partial joint penetration weld of 4.38 mm. The programmed simulation provided realistically accurate results. The difference in weld face width between the simulation and experimental results was 0.16 mm, approximately 0.06 mm in the overlapping zone, and 0.05 mm was the depth difference. Therefore, the programmed heat-source geometry and boundary conditions can be assumed to be accurate [59]. Results of the stress–strain analysis showed that the maximum value of principal stress was equal to 1120 MPa and the total displacement was equal to 0.32 mm. The calculated total displacement maximum value was related to the face of weld geometry; however, for the determined measurement points, the displacement was more than 0.15 mm (Figure 9b, point 3). The maximum principal stress at the measurement points exceeded 250 MPa (Figure 10b, point 4) and was related to sheet restraint, energy dumping factor, thermal gradient resulting from the heat absorbed by welded materials, and the material thermo-mechanical properties [60,61]. The highest displacement value occurred in the fusion zone. The maximum principal stress was related to the fixed geometry.

The simulation and trial joint analysis indicated differences in the cross-sectional hardness distribution, with higher values in the simulation results. The hardness values in BM from the simulation ranged from 181 to 220 HV and varied from the measured values. Moreover, the weld and HAZ achieved higher hardness values in the simulation. The highest value in the weld from the simulation was 237 HV, with the measured value of 231 HV10. In the HAZ, hardness was between 222 and 249 HV, and the measured values ranged from 200 to 218 HV10. The differences may result from the thermal gradient and phase-transformation velocity factor [62]. Moreover, in the numerical simulation, the load applied during the hardness test was not defined. The measured hardness of the trial joint was lower, and the weld zones showed smaller differences in hardness compared to the values calculated in the numerical simulation. The maximum measured value for the trial joint did not exceed 350 HV10, and no additional post-weld heat treatment was carried out.

The tensile strength of the tested joint was 110 MPa, and compared to the BM, it is lower by about 250 MPa. Both tested specimens failed along the fusion zone line at the maximum load of 11.5 kN. During the performed tensile-strength test, according to specimen configuration, both tensile and shear phenomena occurred (Figure 6). Not-uniaxial complex-force distribution affected the test results and the joint strength obtained was related to the tensile–shear strength of the weld. The stress–strain curve did not have the serrated flow region that is characteristic of low-carbon steels, and the plastic–elastic joint character was observed [63,64].

The crystallographic structure of the base material was identified as fine-grained ferritic–pearlitic. The material structure in the fusion zone changed during melting and solidification processes. The metallographic analysis showed a coarse-grained dendritic structure of the weld. Separate dendrite groups formed pillar crystals, with the growth direction related to the fusion line. No impurities or welding defects were detected in the pillar crystals’ contact area. HAZ consist of three areas: the overheated area with a characteristic coarse-grained structure, the normalization area with a uniform fine-grained structure and the partial recrystallization area (incomplete annealing) heated to Ac_1_ ÷ Ac_2_ transformation point during the welding process. The partial recrystallization area consisted of non-transformed ferrite grains and a fine-grained ferritic–pearlitic structure established from the austenite range [65].

The uniform weld structure was analyzed by using ferrite and manganese distributions. The quantity analysis of the distribution of alloying elements in the joint showed a uniform weld structure. Lack of differences along the measurement line of overlap transition (Figure 18 and Figure 19) confirmed the obtainment of a weld of high quality. Ferrite and manganese distribution from the BM to the weld line confirmed a uniform chemical composition of the laser-welded trial lap joint (Figure 16 and Figure 17) [66].

The energy-dispersive X-ray spectroscopy analysis showed some precipitation. No porosity defects were detected in the obtained weld; nevertheless, some oxides in the transition zone were observed. Precipitation in the form of aluminum oxide was detected (Figure 20), which is typical of low-carbon steels and probably related to steel deoxidizing in the metallurgical process, not to the welding process. Further investigations showed other inclusions, in the form of manganese oxide precipitations (Figure 21). The presence of oxides in the weld is related to the absence of shielding gas between the welded steel plates, and the types of oxides are related to the composition of the welded material.

## 5. Conclusions

A numerical simulation of laser welding allowed us to estimate the parameters for the lap joint with partial penetration. By programming properly calibrated heat-source geometry and boundary conditions, accurate results can be obtained. Welding simulation based on thermo-mechanical solution with phase transformation gave realistic results with a convex face of the weld. A single-pass laser-welded lap joint based on calculated parameters was produced, and the properties of the obtained joint were investigated. Hardness in the measured trial joint was lower than that from the calculated results and did not exceed 227 HV10. Therefore, according to restrict B quality level, no additional heat treatment was applied. The tensile test results showed the joint strength was 110 MPa, and it is under nominal strength of BM. The not-uniaxial position resulted in the occurrence of tensile-shearing phenomena. Crystallographic analysis confirmed the typical ferritic–pearlitic structure of the BM, grain refining in the heat affected zone and the weld having a characteristic dendritic structure. No welding defects were detected. The energy dispersive X-ray spectroscopy analysis showed good mixture factor of alloying elements. The fusion zone had a uniform structure. Spectroscopy showed that oxides precipitates in the weld. Manganese oxides were detected in the overlap transition line. No additional shielding gas between welded sheets was used, and atmospheric oxygen affected inclusions. The vacuum atmosphere or complete shielding of the fusion zone by using inert gas could reduce the oxidation process. High-quality welding of the sealed lap joint for a gas pipe system was performed by using a laser beam.

Further research of lap joint welding will consider using twin spot-welding optics for widening the fusion zone, improving strength characteristic and producing a lap joint by using a zigzag-shaped welding trajectory. Further research will apply heat-source enhancement to decrease the differences in hardness values between numerical simulation and measured results. Development of the shielding system for overlap region is planned. Further work on laser lap-joint welding of low-carbon steel based on these assumptions can provide a comprehensive analysis of the investigated problem.

## Figures and Tables

**Table 1:** Chemical composition of low-carbon constructional steel S235JR.

Element	Mn	Si	Cu	Cr and Ni	Nb	Mo	B
Percentage	1.65	0.5	0.4	0.3	0.06	0.08	0.0008

**Table 2:** Thermal properties of low-carbon constructional steel S235JR.

Property	Thermal Conductivity (W/cm·°C)	Specific Heat (J/kg·°C)	Latent Heat (J/g)	Solidus-Liquidus Range (°C)	Austenite Therm. Exp. Coeff. (1/°C)	Ferrite Therm. Exp. Coeff. (1/°C)
**Value**	0.45	480	256	1466.8–1517.1	2.54 × 10^−5^	1.71 × 10^−5^

## References

[1] Steen W.M., Mazumder J. (2010). Laser Material Processing.

[2] Pilarczyk J. (2003). Engineer’s Guide—Welding Engineering.

[3] Unt A., Poutiainen I., Grünenwald S., Sokolov M., Salminen A. (2017). High Power Fiber Laser Welding of Single Sided T-Joint on Shipbuilding Steel with Different Processing Setups. Appl. Sci..

[4] Piekarska W., Kubiak M. (2013). Modeling of thermal phenomena in single laser beam and laser-arc hybrid welding processes using projection method. Appl. Math. Model..

[5] Quiroz V., Gumenyuk A., Rethmeier M. (2012). Laser Beam Weldability of High-Manganese Austenitic and Duplex Stainless Steel Sheets. Weld. World..

[6] Yuce C., Karpat F., Yavuz N. (2019). Investigations on the microstructure and mechanical properties of laser welded dissimilar galvanized steel–aluminum joints. Int. J. Adv. Manuf. Technol..

[7] Gu J., Yang S., Duan C., Xiong Q., Wang Y. (2019). Microstructure and Mechanical Properties of Laser Welded Al-Mg-Si Alloys Joints. Mater. Trans..

[8] Shiegel A.N., Evtikheev N.N., Gusev D.S., Ivanchenko A.B. (2016). Modeling of butt and lap joint welding of aluminum alloys and construction steel sheets. Non-Ferrous Met..

[9] Shanmugam N.S., Buvanashekaran G., Sankaranarayanasamy K. (2013). Experimental Investigation and Finite Element Simulation of laser lap welding of SS304 sheets. Int. J. Mech..

[10] Tasalloti H., Kah P., Martikainen J. (2015). Laser Overlap Welding of Zn-Coated Steel on Aluminium Alloy for Patchwork Blank Applications in the Automotive Industry. Rev. Adv. Mater. Sci..

[11] Oussaid K., El Ouafi A., Chebak A. (2019). Experimental Investigation of Laser Welding Process in Overlap Joint Configuration. Chem. Mater. Sci..

[12] Masoumi M., Marashi S.P.H., Pouranvari M. (2010). Metallurgical and Mechanical Characterization of Laser Spot Welded Low Carbon Steel Sheets. Mater. Technol..

[13] Meco S., Ganguly S., Williams S., McPherson N. (2019). Design of laser welding applied to T joints between steel and aluminium. J. Mater. Process. Technol..

[14] Enz J., Khomenko V., Riekehr S., Ventzke V., Huber N., Kashaev N. (2015). Single-sided laser beam welding of adissimilar AA2024–AA7050 T-joint. Mater. Des..

[15] Kumar S.K. (2015). Numerical Modeling and Simulation of a Butt Joint Welding of AISI316L Stainless Steels Using a Pulsed Laser Beam. Mater. Today Proc..

[16] Meco S., Cozzolino L., Ganguly S., Williams S., McPherson N. (2017). Laser welding of steel to aluminium: Thermal modelling and joint strength analysis. J. Mater. Process. Technol..

[17] Lee C., Lee J.B., Park D.H., Na S.J. (2008). Finite Element Modeling of Laser Spot Welded Lap Joint. Mater. Sci. Forum..

[18] Zhanga Y., Chena Y., Zhoua J., Sunb D., Lib H. (2020). Experimental and numerical study on microstructure and mechanical properties for laser welding-brazing of TC4 Titanium alloy and 304 stainless steel with Cu-base filler metal. J. Mater. Res. Technol..

[19] Ma J., Kong F., Kovacevic R. (2012). Finite-element thermal analysis of laser welding of galvanized high-strength steel in a zero-gap lap joint configuration and its experimental verification. Mater. Des..

[20] Tawfi T.A. (2017). Parametric Optimization of Pulsed Nd:YAG Laser Lap Welding of Stainless Steel ASTM A240/316L with Carbon Steel ASTM A570/Gr30. Al-Nahrain J. Eng. Sci..

[21] Sabbaghzadeh J., Azizi M., Torkamany M.J. (2008). Numerical and experimental investigation of seam welding with a pulsed laser. Opt. Laser Technol..

[22] He Q., Wei H., Chen J.S., Wang H.P., Carlson B.E. (2018). Analysis of hot cracking during lap joint laser welding processes using the melting state-based thermomechanical modeling approach. Int. J. Adv. Manuf. Technol..

[23] Vicentin L.C., Ierardi M.C.F., Garcia A., Vilar R. (2000). Laser welding of low carbon steel blanks. Lasers Eng..

[24] Keskitalo M., Hietala M., Mäntyjärvi K. (2019). The normal and shear strength properties of laser lap weld. Procedia Manuf..

[25] Sindhu R.A., Park M.K., Lee S.J., Lee K.D. (2010). Effects of residual stresses on the static and fatigue strength of laser-welded lap joints with different welding speeds. Int. J. Automot. Technol..

[26] Rizz D., Sibillano T., Calabrese P.P., Ancona A., Lugarà P.M. (2011). Spectroscopic, energetic and metallographic investigations of the laser lap welding of AISI 304 using the response surface methodology. Opt. Lasers Eng..

[27] Meco S., Pardal G., Ganguly S., Williams S., McPherson N. (2015). Application of laser in seam welding of dissimilar steel to aluminium joints for thick structural components. Opt. Lasers Eng..

[28] Evdokimov A., Springer K., Doynov N., Ossenbrink R., Michailov V. (2017). Heat source model for laser beam welding of steel-aluminum lap joints. Int. J. Adv. Manuf. Technol..

[29] Ki H., Mazumder J., Mohanty P.S. (2002). Modeling of laser keyhole welding: Part, I. mathematical modeling, numerical methodology, role of recoil pressure, multiple reflections, and free surface evolution. Metall. Mat. Trans. A.

[30] Fey A., Ulrich S., Jahn S., Schaaf P. (2020). Numerical analysis of temperature distribution during laser deep welding of duplex stainless steel using a two-beam method. Weld. World.

[31] Bag S., Kiran D.V., Syed A.A., De A. (2012). Efficient Estimation of Volumetric Heat Source In Fusion Welding Process Simulation. Weld. World.

[32] Wang R., Lei Y., Shi Y. (2011). Numerical simulation of transient temperature field during laser keyhole welding of 304 stainless steel sheet. Opt. Laser Technol..

[33] Ribic B., Rai R., DebRoy T. (2008). Numerical simulation of heat transfer and fluid flow in GTA/Laser hybrid welding. Sci. Technol. Weld. Join..

[34] Danielewski H. (2019). Laser welding of pipe stubs made from super 304 steel. Numerical simulation and weld properties. Tech. Trans..

[35] Kik T. (2020). Computational Techniques in Numerical Simulations of Arc and Laser Welding Processes. Materials.

[36] Kumar U., Gope D.K., Srivastava J.P., Chattopadhyaya S., Das A.K., Krolczyk G. (2018). Experimental and Numerical Assessment of Temperature Field and Analysis of Microstructure and Mechanical Properties of Low Power Laser Annealed Welded Joints. Materials.

[37] Chunming W., Bin L., Ping J., Xiang X., Gaoyang M. (2018). Numerical and experimental investigation of vacuum-assisted laser welding for DP590 galvanized steel lap joint without prescribed gap. Int. J. Adv. Manuf. Technol..

[38] Kubiak M., Piekarska W., Stano S., Saternus Z. (2015). Numerical modelling of thermal and structural phenomena in Yb:Yag laser butt-welded steel elements. Arch. Metall. Mater..

[39] Yilbas B.S., Arif A.F.M., Abdul Aleem B.J. (2010). Laser welding of low carbon steel and thermal stress analysis. Opt. Laser Technol..

[40] Podany P., Reardon C., Koukolikova M., Prochazka R., Franc A. (2018). Microstructure, Mechanical Properties and Welding of Low Carbon, Medium Manganese TWIP/TRIP Steel. Metals.

[41] Nagel F., Simon F., Kümmel B., Bergmann J.P., Hildebrand J. (2014). Optimization Strategies for Laser Welding High Alloy Steel Sheets. Phys. Procedia.

[42] Andersson O., Budak N., Melander A., Palmquist N. (2017). Experimental measurements and numerical simulations of distortions of overlap laser-welded thin sheet steel beam structures. Weld. World.

[43] Hatami N., Babaei R., Dadashzadeh M., Davami P. (2008). Modeling of hot tearing formation during solidification. J. Mater. Process. Technol..

[44] Hafez K.M., Ramadan M., Fathy N., Ismail M. (2017). Microstructure and Mechanical Properties of Laser Welded Dual Phase and Mild Steel Joints for Automotive Applications. Appl. Mech. Mater..

[45] Górka J., Stano S. (2018). Microstructure and Properties of Hybrid Laser Arc Welded Joints (Laser Beam-MAG) in Thermo-Mechanical Control Processed S700MC Steel. Metals.

[46] Zdravecka E., Slota J. (2019). Mechanical and Microstructural Investigations of the Laser Welding of Different Zinc-Coated Steels. Metals.

[47] PN-EN ISO 6507-1:2018 (2018). Metallic Materials—Vickers Hardness Test—Part 1: Test Method.

[48] PN-EN ISO 9018:2003 (2003). Destructive Tests on Welds in Metallic Materials—Tensile Test on Cruciform and Lapped Joints.

[49] PN-EN ISO 17639:2003 (2003). Destructive Tests on Welds in Metallic Materials—Macroscopic and Microscopic Examination of Welds.

[50] Yan F., Fang X., Chen L., Wang C., Zhao J., Chai F., Wang W. (2018). Microstructure evolution and phase transition at the interface of steel/Al dissimilar alloys during Nd: YAG laser welding. Opt. Laser Technol..

[51] David S.A., Vitek J.M. (1989). Correlation between solidification parameters and weld microstructures. Int. Mater. Rev..

[52] Partes K., Schmidt M., Gorny S. (2020). Prediction of Preheating Temperatures for S690QL High Strength Steel Using FEM-Simulation for HighPower Laser Welding. Lasers Manuf. Mater. Process..

[53] Sun J., Liu X., Tong Y., Deng D. (2014). A comparative study on welding temperature fields, residual stress distributions and deformations induced by laser beam welding and CO2 gas arc welding. Mater. Des..

[54] Kang M., Jeon I.-H., Han H.N., Kim C. (2018). Tensile–Shear Fracture Behavior Prediction of High-Strength Steel Laser Overlap Welds. Metals.

[55] Sinha A.K., Kim D.Y., Ceglare D. (2013). Correlation analysis of the variation of weld seam and tensile strength in laser welding of galvanized steel. Opt. Lasers Eng..

[56] Hietala M., Järvenpää A., Keskitalo M., Jaskari M., Mäntyjärvi K. (2019). Tensile and fatigue properties of laser-welded ultra-high-strength stainless spring steel lap joints. Procedia Manuf..

[57] Evin E., Tomas M. (2017). The Influence of Laser Welding on the Mechanical Properties of Dual Phase and Trip Steels. Metals.

[58] Gorka J. (2011). Properties of thermomechanically treated welds of high yield point steel. Weld. Technol. Rev..

[59] Lee K.D., Ho K.I., Park K.Y. (2014). Analysis of the Local Stresses at Laser Welded Lap Joints. Weld. J..

[60] Deng D. (2009). FEM prediction of welding residual stress and distortion in carbon steel considering phase transformation effects. Mater. Des..

[61] Hsu C., Albright C.E. (1991). Fatigue analysis of laser welded lap joints. Eng. Fract. Mech..

[62] Salminen A., Baskutis S., Petronis E. (2017). Influence of welding modes on weldability of structural steel lap joints in laser welding. J. Laser Appl..

[63] Goyal R., El-zein M. (2020). Influence of laser weld shape on mechanical and fatigue behaviour of single lap laser welded joints. J. Adv. Join. Process..

[64] Furusako S., Miyazaki Y., Akiniwa Y. (2015). Tensile shear strength of laser lap joints. Weld. Int..

[65] Farabi N., Chen D.L., Li J., Zhou Y., Dong S.J. (2010). Microstructure and mechanical properties of laser welded DP600 steel joints. Mater. Sci. Eng. C.

[66] Zhang C., Li G., Gao M., Zeng X.Y. (2017). Microstructure and Mechanical Properties of Narrow Gap Laser-Arc Hybrid Welded 40 mm Thick Mild Steel. Materials.

